# Contrast-medium administration for prostate MRI: yes! Contrast-medium administration can be abandoned

**DOI:** 10.1007/s00330-023-09766-y

**Published:** 2023-07-07

**Authors:** Patrick Asbach

**Affiliations:** Department of Radiology, Charité – Universitätsmedizin Berlin, Berlin, Germany

Over the past few years, multiparametric MRI (mpMRI) of the prostate has emerged as the most successful radiologic imaging study for detecting prostate cancer. The PI-RADS system and the MRI pathway have paved the way for a widespread usage of this imaging test in daily clinical practice [1]. Prostate MRI is on the verge of opening up further indications towards screening, which is quite remarkable. As a result, the number of prostate MR scans has increased sharply and this trend will continue in the next years. This puts pressure on the justification to perform the most invasive and “controversial” part of MRI of the prostate, the dynamic contrast-medium-enhanced sequence. It is controversial because it is considered an integral part of prostate MRI on the one hand but apparently has no major impact since it translates into a change in the PI-RADS score in only one out of 10 scenarios (peripheral zone PI-RADS 3 lesion on the dominant sequence). In fact, the large prospective trials that have gathered the body of evidence for making prostate MRI into all the important urology guidelines are based on mpMRI. What would be the likely consequences of skipping the dynamic contrast-enhanced sequence in favor of a biparametric MRI (bpMRI) approach? One consequence would be a slightly lower sensitivity (and lower negative predictive value) for the detection of clinically significant prostate cancer [2], but one can suspect most missed cancers will be Gleason 3 + 4 = 7a cancers which cause minimal harm by delayed diagnosis [3]. It would also result in some lack of imaging information in patients whose scans have a lower image quality related to artifacts mostly on the diffusion-weighted sequence (DWI) related to, e.g., hip replacements; however, the gradient echo sequence commonly used for dynamic contrast-enhanced imaging is also susceptible to artifacts [4]. Readers with lesser experience potentially miss a lesion if no contrast-enhanced sequence is available to them, but it is less likely that they overcall focal prostatitis as cancer. bpMRI can to some extent compensate the lack of contrast-medium administration for example by rigorous image quality assurance and double-reading with experts (or AI tools) for lesser experienced radiologists. Also, accounting PSA density as an additional parameter might trigger follow-up imaging in patients at risk which would benefit from safety-netting. Many urologists will probably biopsy a lesion no matter whether it is PI-RADS 3 or 4; the information they need from the MRI scan is very simple. This information that a radiologist ultimately delivers to the urologist because he or she requested the prostate MRI can be, in the prostate cancer detection scenario, condensed to as follows: (i) is there a lesion that a targeted biopsy should be done on “yes or no,” and (ii) if so where is it located within the prostate. In fact, two meta-analyses on the diagnostic performance of bpMRI revealed a pooled sensitivity for clinically significant prostate cancer detection of 0.87 (0.83), specificity of 0.72 (0.71), and an AUC value of 0.87 (0.84) [2, 5]. A meta-analysis on head-to-head comparisons of bpMRI versus mpMRI showed a pooled sensitivity and specificity of 0.74 and 0.90 for bpMRI versus 0.76 and 0.89 for mpMRI [6], respectively. These data are very promising, but higher-quality level data are ultimately needed until the PI-RADS committee can give a definite statement on bpMRI because all comparative studies have used multiparametric MRI for biopsy decisions [7]. The two ongoing prospective clinical trials PRIME (PRostate Imaging using Mri ± contrast Enhancement—NCT04571840) [8] and PACIFIC (Prostate Assessment using Comparative Interventions – Fast mri and Image-fusion for Cancer—NCT05574647) [9] which assess if bpMRI is non-inferior to mpMRI for the detection of clinically significant prostate cancer will provide the final piece of evidence to ultimately justify abandoning contrast-medium application in prostate MRI. The patients, the hospital administrators that often push towards faster scanning, the environment [10], and many radiologists who have to assess hundreds of images will appreciate that.

## Declarations

### Guarantor

The scientific guarantor of this publication is Patrick Asbach.

### Statistics and biometry

No complex statistical methods were necessary for this paper.

### Study subjects or cohorts overlap

N/A
